# Nutritional Strategies to Combat Type 2 Diabetes in Aging Adults: The Importance of Protein

**DOI:** 10.3389/fnut.2019.00138

**Published:** 2019-08-28

**Authors:** Kayleigh M. Beaudry, Michaela C. Devries

**Affiliations:** Department of Kinesiology, Faculty of Applied Health Sciences, University of Waterloo, Waterloo, ON, Canada

**Keywords:** protein, dairy, aging, type 2 diabetes, sarcopenia, resistance exercise

## Abstract

The prevalence of pre-diabetes (PD) and type II diabetes (T2D) has risen dramatically in recent years affecting an estimated 422 million adults worldwide. The risk of T2D increases with age, with the sharpest rise in diagnosis occurring after age 40. With age, there is also a progressive decline in muscle mass starting after the age of 30. The decline in muscle mass and function due to aging is termed sarcopenia and immediately precedes the sharp rise in T2D. The purpose of the current review is to discuss the role of protein to attenuate declines in muscle mass and insulin sensitivity to prevent T2D and sarcopenia in aging adults. The current recommended dietary allowance for protein consumption is set at 0.8 g/kg/day and is based on dated studies on young healthy men and may not be sufficient for older adults. Protein consumption upwards of 1.0–1.5 g/kg/day in older adults is able to induce improvements in glycemic control and muscle mass. Obesity, particularly central or visceral obesity is a major risk factor in the development of PD and T2D. However, the tissue composition of weight loss in older adults includes both lean body mass and fat mass and therefore may have adverse metabolic consequences in older adults who are already at a high risk of lean body mass loss. High protein diets have the ability to increase weight loss while preserving lean body mass therefore inducing “high-quality weight loss,” which provides favorable metabolic changes in older adults. High protein diets also induce beneficial outcomes on glycemic markers due to satiety, lowered post-prandial glucose response, increased thermogenesis, and the ability to decrease rates of muscle protein breakdown (MPB). The consumption of dairy specific protein consumption has also been shown to improve insulin sensitivity by improving body composition, enhancing insulin release, accelerating fat oxidation, and stimulating rates of muscle protein synthesis (MPS) in older adults. Exercise, specifically resistance training, also works synergistically to attenuate the progression of PD and T2D by further stimulating rates of MPS thereby increasing muscle mass and inducing favorable changes in glycemic control independent of lean body mass increases.

## Introduction

The average lifespan has increased by 20 years, increasing the age of the population worldwide (1). At the same time, the rates of obesity have also increased radically (2). In 2015, 603.7 million adults were considered obese, which is nearly double that reported in 1980 (2). Obesity is associated with a multitude of co-morbidities such as cardiovascular disease, metabolic syndrome, and type 2 diabetes (T2D) (2, 3). The prevalence of pre-diabetes (PD) and T2D has risen dramatically in recent years affecting an estimated 422 million adults worldwide (4). T2D is characterized by elevated blood glucose levels caused by an impairment in glucose tolerance due to the development of insulin resistance (IR) and relative insulin deficiency (5). IR and impaired insulin sensitivity (IS) decrease the ability of muscle cells to take up and store glucose (5).

The risk of T2D increases with age, with the sharpest rise in diabetes diagnosis occurring after age 40 (6). With age there is a progressive loss of muscle mass and strength, termed sarcopenia, that begins in the fourth decade (7). It is predicted that muscle mass loss progresses at a rate of 3–8% per decade beyond age 30 (7), which immediately precedes the sharp rise in T2D incidence (7, 8). Sarcopenia is a syndrome that is characterized by progressive and generalized loss of skeletal muscle mass and strength and may lead to physical disability, poor quality of life, loss of autonomy, as well as death (9, 10). Sarcopenia can affect up to 45% of men and 26% of women in the general population (11). The risk of sarcopenia is greater in individuals with T2D because they have decreased muscle strength, muscle mass and muscle quality compared to healthy age-matched controls (12–16). Decreases in muscle mass have huge implications for glucose handling as muscle mass is the largest storage depot for glucose in the body, accounting for >75% of glucose disposal (17); thus as it is lost there is a corresponding decrease in glucose storage capacity. While both diabetes and sarcopenia are affiliated, their interaction is not fully understood (12), with sarcopenia being a potential cause and/or consequence of T2D (18).

Lifestyle factors such as diet and exercise play a crucial and central role in glucose handling and IS (19). While exercise has long been recognized for its glucose sensitizing effects, the ability to exercise in older populations may be compromised by frailty, physical disability, and disease (20). Meanwhile, diet also plays a substantial role in the prevention and treatment of both diabetes and sarcopenia. Specifically, dietary protein consumption and dietary-derived amino acids may be the greatest alternative to slow or prevent muscle protein catabolism in older adults (21, 22). With age however, there is a decrease in protein consumption and efficiency that may be due to decreased appetite, difficulty in mastication or changes in digestion (23). The purpose of the current review is to discuss the role of protein to attenuate declines in muscle mass and insulin sensitivity to prevent T2D and sarcopenia in aging adults.

## Role of Muscle Mass in the Regulation of Plasma Glucose and Maintenance of Insulin Sensitivity

### Muscle Mass, Aging, and Insulin Resistance

Muscle mass is important beyond its role to promote movement of the human body. Skeletal muscle also contributes significantly to postprandial glucose disposal, lipid oxidation, resting metabolic rate, and whole-body protein metabolism (24, 25). Muscle mass is determined by the relative rates of muscle protein synthesis (MPS) and muscle protein breakdown (MPB). In the fasted state, rates of MPB exceed MPS, resulting in negative protein balance in skeletal muscle (26). In response to protein feeding there is a significant increase in the rate of MPS due to increased amino acid availability and a reduction in the rate of MPB due to increased circulating insulin, which results in a state of net positive protein balance (27). Overall net protein balance is determined by the relative rates of MPS and MPB during these fed and fasted periods, which over time will dictate whether there is a gain, loss, or maintenance of skeletal muscle mass (25, 28).

The progressive loss of lean body mass that occurs with aging is due to an imbalance between MPS and MPB (22, 29). While earlier studies reported that age-related muscle mass loss could be due to a decline in basal rates of MPS (30–32), elevated rates of MPB (33) or a combination of both, recent evidence suggests that it is due to a blunted MPS response to protein feeding, termed anabolic resistance (34–37). Indeed, a study by Volpi et al. (34) found that when infusing both young and old men and women with an amino acid and glucose mixture, rates of MPS were elevated only in the young healthy adults. These findings were confirmed by Cuthbertson et al. (36) who found no differences in MPS rates between young and older men at rest, but the MPS response to a bolus of crystalline essential amino acids was attenuated in older as compared with younger men. Similarly, Smith et al. (38) found similar basal MPS rates between young and old men and a blunted MPS response to an amino acid infusion in older men. However, this study also found that basal MPS rates were ~30% higher in old as compared with young women and that in response to the amino acid infusion MPS increased in young, but not older women (38). Together these findings suggest that there is unlikely a deficit in basal rates of MPS in older adults, but rather a decreased sensitivity and responsiveness of MPS to feeding stimuli. Furthermore, these findings suggest that there is a sexual dimorphic effect of aging on basal and fed rates of MPS.

**Figure 1:**
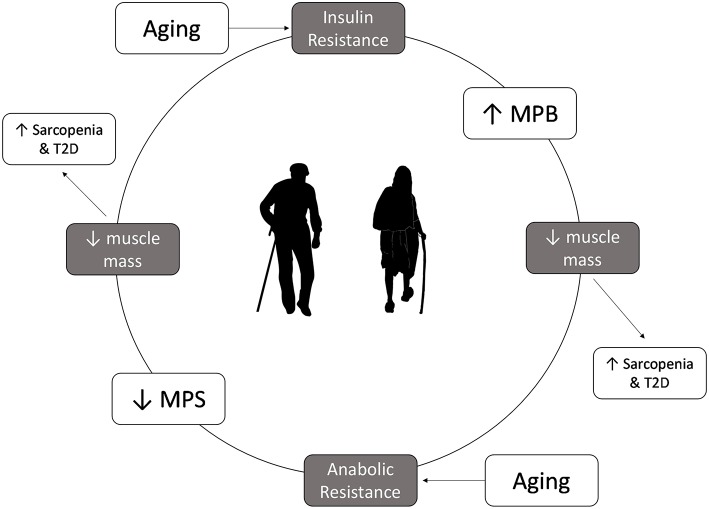
Insulin resistance and anabolic resistance are the hallmarks of aging and can exacerbate the increased risk of sarcopenia and type 2 diabetes. Insulin resistance results in a lower ability to decrease muscle protein breakdown in the fed state, which can lead to loss of muscle mass, contributing to development of sarcopenia. Older adults have anabolic resistance, which leads to decrease in muscle protein synthesis in the fed state, which overtime leads to muscle mass loss. Lower muscle mass will lower glucose storage capacity, which in turn can increase the risk of type 2 diabetes.

Muscle mass loss in aging is worsened in disease states characterized by IR, such as T2D and PD (Figure 1). In healthy adults, insulin helps regulate protein metabolism in muscle and is essential for muscle growth (9). Furthermore, while insulin plays a permissive role in promoting MPS in the presence of amino acids, it is essential to allow for the reduction of MPB in the fed state (27). In the IR state insulin is unable to reduce MPB in the fed state, ultimately leading to an even more negative muscle protein balance (9), breakdown of muscle protein and muscle wasting (39). As such, adults who are IR are at an even greater risk for sarcopenia as they age as they not only are less able to mount an anabolic response to protein feeding, but they are less able to blunt MPB in the fasted state. Indeed, studies in older pre-diabetic or diabetic individuals show that rates of muscle mass decline are greater than that seen in healthy, older adults (40) and that they have lower muscle mass, strength and function than age-matched controls (41).

Skeletal muscle is the largest storage depot for glucose in the body, accounting for >75% of glucose disposal (17, 42). In fact, skeletal muscle mass relative to body weight has been shown to be inversely related with IR (42). As such, the reduction in muscle mass seen with aging may adversely influence IR and the risk of developing T2D. These findings suggest that even healthy older adults are living in a vicious cycle whereby muscle mass loss can lead to IR, which can lead to further muscle mass loss. Furthermore, those who begin the aging process with impaired IS, PD, or T2D may be at an even greater risk for sarcopenia as they have entered this cycle earlier and are losing muscle mass at a greater rate (40). However, strategies that can enhance muscle mass may have a profound effect on preventing the development of T2D in older adults, as research has shown that even a 3% incremental increase in muscle mass is associated with a 7.5% relative reduction in prediabetes risk (42).

## Nutritional Strategies in Aging, Sarcopenia, Type 2 Diabetes

### Protein Recommendations for Older Adults and Individuals With Type 2 Diabetes

Currently the recommended dietary allowance (RDA) for protein consumption is 0.8 g/kg/day of protein or 10–35% of total energy intake (11, 43, 44). However, this recommendation was based primarily on the dated nitrogen balance method and studies performed in young healthy men and may not cover the needs of aging older adults (11). Indeed, there is a great deal of evidence advocating for higher daily protein intakes of 1.0–1.2 g/kg/day in healthy older adults, particularly those who require additional support, to preserve muscle mass and function (29, 45, 46). Further recommendations include an increased amount of 1.2–1.5 g/kg/day in older adults who are malnourished or at a risk of malnutrition with even higher recommendations for individuals with severe illness or injury (46). Complicating matters is the finding that older adults are less likely to consume adequate protein amounts compared to their younger counterparts (47). Unfortunately, approximately one third of adults over the age of 50 are not meeting the protein RDA, with a staggering 35% of older adults in institutional care who are not meeting the estimated average requirement (EAR), which is the minimum intake level of protein to maintain proper muscle integrity (43, 48), which may be affecting their overall health and disease risk.

Although lifestyle management such as a healthy diet has long been recommended to improve glycemic control, it is not certain what dietary approach is best for individuals with diabetes with most recommendations centered around individualized needs based on glycemic control, age, and co-morbidities (44). There remains some debate about what constitutes the ideal macronutrient composition for a healthy diet for diabetes (44). While there are dietary recommendations for individuals with PD or T2D, the focus remains on improving glycemic control through reducing energy intake, reducing dietary fat and saturated fat intake, and increasing dietary fiber intake (44, 49). In fact, protein intake recommendations do not differ from that recommended for the general population, despite protein anabolic resistance and a greater need for protein as a result of inflammation and oxidative modification of proteins in individuals with diabetes (50).

**Figure 2:**
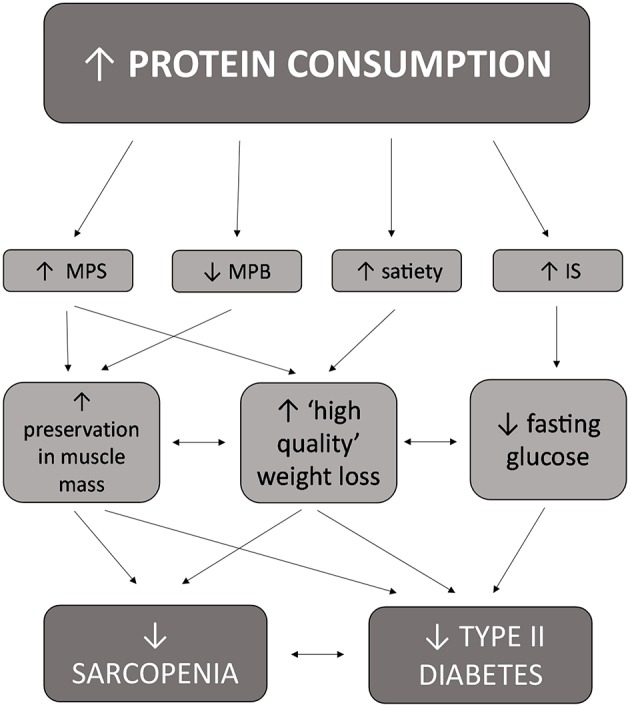
Interrelated effects of increased protein consumption on factors related to both sarcopenia and type II diabetes in older adults.

Historically, there were several reasons why higher protein diets were not recommended for older adults and/or individuals with metabolic alterations including T2D, including the thought that dietary protein would adversely raise blood glucose levels and have a detrimental effect on kidney function (44). The belief that dietary protein is converted into glucose upon consumption and adversely increases blood glucose concentrations is thought to have originated from a study by Janney conducted in 1915 where 3.5 g of glucose was produced from consuming 6.25 g of protein from meat (51). These findings have been discredited by several studies, the first of which was conducted in 1924 involving participants with and without diabetes who were fed 50 g of protein and showed no change in blood glucose concentrations (52). Furthermore, a study conducted in 1936 that found that consuming even large amounts of protein (1.3 pounds) in a single serving does not raise blood glucose concentrations (53). This remains true in individuals with diabetes and impaired glucose control, with dietary protein exhibiting insulinotropic effects and enhancing blood glucose clearance from the blood (54–56). The second reason why higher protein intakes were not encouraged for individuals with diabetes pertains to the theory that increased dietary protein intake would lead to kidney disease, a theory that has also been discredited. A recent meta-analysis conducted by Devries et al. (57) indicated that high protein diets (≤1.5 g/kg/ body weight or ≤20% energy intake or ≤100 g protein per day) did not adversely influence kidney function on glomerular filtration rate in adults without kidney disease. Furthermore, a sub-analysis revealed that increased protein consumption did not adversely affect kidney function in individuals with type 2 diabetes (57). More recent evidence now supports a positive effect of a protein-rich diet in diabetes (44) and sarcopenia (11). These positive outcomes are thought to be due to several mechanisms, including an increase in protein anabolism, weight loss, enhanced glycemic control, daily appetite control, and satiety (47, 58) (Figure 2).

### Protein Intake and Its Effect on Insulin Sensitivity and Glycemic Control

Obesity, particularly central or visceral obesity, is a risk factor for the development of T2D and is common in PD and T2D (59). Weight loss remains a dominant determinant of reducing the risk of diabetes, with a weight loss of ~5 kg accounting for an ~55% reduction in the risk of T2D in overweight or obese individuals with impaired glucose control (60). Weight loss, even modest weight loss in overweight and obese subjects has been shown to improve markers of glycemic control in T2D (61–63). Energy-restricted high protein, low carbohydrate diets have been successful in improving weight loss and glucose control in T2D (49). However, it is not just the amount of weight loss that is important, but also the composition of weight loss. While traditional energy restriction leads to weight loss, this type of weight loss induces not only a loss of fat mass (~75% of mass lost), but also a loss of muscle mass (~25% of mass lost), which could have adverse metabolic consequences particularly in older adults who are already losing muscle mass (64–66). In fact, it has been shown that in older adults, total weight loss was represented by a higher proportion of lean mass loss compared to fat mass, whereas weight gain was largely represented by an increase in fat mass (67). This decrease in lean body mass can have adverse metabolic consequences and accelerate the development of T2D and sarcopenia in older adults.

Higher protein diets may be especially important to improve IS in older adults with PD or T2D as higher protein intakes during weight loss can help preserve muscle mass (66, 68), inducing what is known as “high-quality” weight loss. A study by Piatti et al. (69) found that while hypocaloric high protein and high carbohydrate diets had similar effects on the total amount of weight loss, the high protein diet was able to improve insulin sensitivity and spare lean body mass whereas the high carbohydrate diet did not. Furthermore, a study by Wycherley et al. (70) found that as compared with a standard protein diet, a high protein diet induced a greater reduction in fat mass (2.1 kg) in men and women with T2D. Glycemic control and insulin concentrations improved in all groups with no difference between groups; however, the change in insulin concentration was related to the extent of fat mass loss, suggesting that a higher protein diet may exert a more favorable effect on glycemic control than a standard protein diet (70). Promotion of “high quality” weight loss may be particularly important in older adults who are already losing muscle mass (16, 66). By inducing energy restriction while increasing or maintaining the consumption of protein at 1.6 g/kg/day or 30% of energy intake, the amount of lean mass that is lost is reduced (71, 72). The sparing of lean muscle mass is an important aspect to consider as skeletal muscle mass is an extremely metabolically active tissue and the loss of lean tissue mass may be in part responsible for the plateau in weight loss or weight regain following weight loss programs (73, 74).

In addition to inducing “high-quality” weight loss, energy restricted, higher protein diets have also been found to induce greater weight loss than traditional low-fat, standard energy restricted diets (75), likely at least in part, due to the effects of protein consumption on satiety. Satiety or the perceived feeling of fullness after a meal has been shown to be significantly higher following high protein meals (47). Post-prandially there are reductions in perceived hunger and increases in perceived fullness after consuming a high protein meal compared to a standard protein meal (47). When dietary protein intake is increased from 15 to 30% of energy intake and carbohydrate remains constant there is a decrease in *ad libitum* caloric intake due to increased leptin sensitivity (76). Mechanistically this is due to the effect of protein consumption on the gut hormone response. Specifically, after the consumption of a high protein meal there are reductions in the hunger stimulating hormone ghrelin as well as increases in the satiety-stimulating hormones PYY and GLP-1 (47, 77). The impact of protein consumption on satiety is significant as higher protein diets may help regulate appetite control, satiety and prevent increased caloric intake and overeating, which can help facilitate weight loss and/or weight maintenance.

Higher protein diets also assert beneficial effects on IS and glycemic control independent of weight loss. A study by Gannon and Nuttall (78) found that in diabetic men after 5 weeks on a high protein, low carbohydrate diet there was a decrease in fasting blood glucose levels and glycohemoglobin content with no significant changes in body weight. Another study from the same research group confirmed these findings by showing that a high protein, low carbohydrate diet lowered the postprandial glucose response and improved overall glucose control in diabetic men and women despite no changes in body weight compared to a more traditional high carbohydrate diet (79). Together these studies suggest that consuming a higher protein diet is beneficial to improve glycemic control in individuals with T2D during periods of weight maintenance. Considering that weight loss is not always recommended in older adults due to the effects of weight loss on lean body mass (67, 80), these findings are important as they suggest that a higher protein diet may be efficacious to improve glycemic control in older individuals with IR, PD, or T2D without weight loss; however, studies in IR older adults are required.

### The Special Case for Dairy-Based Protein Consumption to Improve Muscle Mass and Insulin Sensitivity in Older Adults

The consumption of dairy products has also has been shown to improve IS (81). A study by Choi et al. (82) found that each additional serving per day of dairy was associated with a 9% lower risk of T2D in men. The protective effect of dairy intake was seen regardless of body mass index, physical activity levels, and family history (82). This relationship has been confirmed in women irrespective of age, BMI, physical activity level, and family history, with each additional dairy serving per day associated with a 4% lower risk for T2D (83). Furthermore, an inverse relationship between frequency of dairy intake and insulin resistance syndrome (IRS) in overweight adults was also observed in the CARDIA study (84). IRS was defined as the presence of 2 or more of abnormal glucose homeostasis, obesity, elevated blood pressure, and dyslipidemia, all which increase the risk of developing T2D. In this study the 10-year incidence of IRS was two thirds lower in overweight adults who consumed more than 5 servings/day of dairy products compared to those who consumed <1.5 servings/day (84).

Results from prospective trials examining whether increasing dairy consumption can enhance IS have been promising. A study by Rideout et al. (81) found that consumption of 4 servings/day of low-fat dairy milk and yogurt products reduced fasting plasma insulin by 9% and improved IR by 11% in overweight and obese adults over a 6-month time period. Yogurt may be especially effective at enhancing IS due to its probiotic content. Certain species of probiotics have been found to prevent weight gain, prevent obesity, improve energy metabolism, and enhance insulin sensitivity (85). Several studies have shown that the consumption of probiotic yogurt reduces fasting blood glucose concentration and glycosylated hemoglobin levels in patients with T2D (86) and induces positive changes in lipid profiles and insulin sensitivity (85). This suggests that the composition and/or diversity of the gut microbiota may contribute to the development of T2D and that supplementing with probiotics could be useful in preventing IR (87). A protective effect of probiotic cultures on gut permeability and gut barrier function is one potential mechanism that has been suggested to explain the positive effects of probiotic yogurt on IR (87). Decreases in gut barrier function may be linked to diet-induced changes that lead to the development of IR and T2D in animal models by increased endotoxemia which allows harmful macromolecules and microorganisms through the barrier (88). Taken together, the results from these studies suggest that increasing dairy consumption, particularly yogurt, may help in the prevention and management of T2D.

There are several potential mechanisms by which increased dairy intake can improve IS, thus preventing T2D. The milk proteins, casein and particularly whey, have insulinotropic properties, meaning they enhance the release of insulin, while maintaining a low glycemic response (83, 89). In a study by Pal et al. (90), 12 weeks of whey protein supplementation (54 g/day) decreased fasting plasma insulin levels by 11% and IR by 10% in overweight and obese older adults. Similarly, in T2D men and women, when whey protein was added to the breakfast and lunch meals there was a 31 and 57% increased insulin response after breakfast and lunch, respectively, which resulted in a lower blood glucose response to the lunch meal (91). Mechanistically this may be due to the effect of whey to increase incretin secretion. In particular, whey protein is a potent stimulus for the secretion of glucagon-like peptide (83) and glucose-dependent insulinotropic polypeptide (91), both of which stimulate insulin secretion and inhibits glucagon secretion, inhibiting hepatic glucose production and thereby lowering blood glucose concentrations (92).

Dairy products are also excellent sources of magnesium, calcium, lactose, and dairy protein, which have been shown to increase satiety, which may protect against weight gain and obesity (82–84) and promote greater weight loss during energy restriction (93). Vitamin D is also recognized for its insulin sensitizing effect through regulation of insulin receptor expression and stimulation of insulin release by the beta-cells of the pancreas (94, 95). Together, these data support a role for multiple components of dairy products to work synergistically to enhance IS through different mechanisms, decreasing the risk of developing T2D.

Of particular pertinence to older adults with IR, PD, or T2D, increased dairy consumption has also been found to induce favorable effects on body composition (8). Dairy products contain high levels of calcium, which has been shown to accelerate fat loss, while maintaining lean body mass, by increasing fecal fat excretion (96), decreasing fat absorption (97), increasing fat oxidation (98) and increasing the thermic effect of food (99). Furthermore, dairy products are an excellent source of whey protein and whey protein consumption leads to greater increases in MPS than other proteins due to its rich leucine content (90, 100), suggesting that it may induce favorable effects on lean body mass. Indeed, whey protein has been shown to enhance lean mass in numerous populations including young (101), older adults (102), and PD/T2D (103) as well as preserve muscle anabolism and lean body mass during weight loss (104, 105). These findings suggest that increased dairy consumption may promote “high quality” weight loss, which as detailed above, would be favorable for the prevention and management of T2D in older adults. Indeed, a study by Josse et al. (66) in overweight and obese, premenopausal women found significantly greater fat loss with a gain in lean body mass during a 16-week hypo-energetic diet with 30% of energy intake from dietary protein, with one half of the total protein intake from dairy sources. Higher intakes of dairy protein combined with an aerobic exercise intervention 7 day/week with an additional 2 day/week of resistance training, induced greater total and visceral fat losses and greater lean mass gains compared to those who consumed diets lower in protein lacking dairy foods (66). Furthermore, a recent meta-analysis of 27 randomized controlled trials found that consumption of 2–4 servings/day of dairy foods or 20–84 g/day of whey protein resulted in a greater loss of body weight and fat mass and a smaller loss of lean mass as compared with low dairy control diets (93). Increased dairy consumption may therefore be particularly beneficial for older adults with impaired glucose control like PD and T2D due to the combined effect of dairy on both IS and lean body mass.

## Exercise, Type 2 Diabetes, and Sarcopenia

Exercise has long been known for its ability to decrease or attenuate the progression of PD and T2D (19, 106). Exercise induces positive effects on glucose handling in both healthy individuals and those with impaired glucose handling (19, 107–109), such that a single bout of exercise can markedly increase post-exercise glucose control up to 20-fold for up to 72 h, depending on exercise type, intensity and duration (19, 107, 109–112). Although the beneficial effects of exercise are well-known in relation to T2D and all-cause mortality, individuals with T2D are among the least likely population to exercise and the adherence rate of physical activity are exceptionally low (113). Some barriers to exercise in these individuals include poor health, lack of company, lack of interest and lack of time (113). While increasing physical activity and/or exercise is considered a fundamental treatment for the prevention and management of T2D and the benefits of aerobic exercise are well-known (109, 114), we are going to focus on the effects of resistance exercise on IS since it is the most efficacious exercise strategy to improve muscle mass and thus may be the most effective strategy to prevent sarcopenia and T2D.

### Protein Enhances the Effects of Resistance Exercise on Muscle Mass and IS

Resistance exercise is the primary mode of exercise to elicit positive changes in muscle mass (115) by significantly increasing the rate of MPS (25), which over time leads to muscle hypertrophy. A recent meta-analysis found that resistance training increases lean body mass by ~1 kg in older adults (116). While this 1 kg increase may appear modest, this increase is in contrast to the ~0.18 kg/year decline in lean body mass that occurs beyond the age of 50 (116). Although the effect of resistance training alone may not be enough to promote a net positive protein balance, when protein is consumed after a bout of resistance exercise rates of MPS can be elevated for up to 24 h (117), which may attenuate the decline in lean body mass in older adults. Indeed, while 24 weeks of resistance training in frail older adults improved muscle strength and functional performance, only the group supplemented with protein also had a significant increase in skeletal muscle mass (118). Therefore, it may be the synergistic approach of repeated bouts of resistance exercise and protein consumption that results in the greatest skeletal muscle hypertrophy (119). Support for this theory comes from a recent meta-analysis that found that protein consumption during resistance training induced a 0.3 kg greater increase in lean body mass in young and older adults (120). Thus, the combination of resistance exercise and protein consumption may be especially beneficial for older adults with IR, PD, or T2D to attenuate declines in lean body mass.

Resistance exercise can also directly improve glycemic control through several mechanisms including, (1) increasing muscle mass, which in turn will increase glucose storage capacity, (2) upregulating insulin signaling proteins, and (3) inducing GLUT 4 translocation to the cell membrane to facilitate glucose clearance from circulation during and immediately after exercise (121). While high protein diets alone induce improvements in body composition and IS in older adults with impaired glucose control (69, 122), the addition of resistance exercise exerts an added benefit on IS and glucose handling. A study by Castaneda et al. (123) involving 16 weeks of resistance training in older men and women with T2D, found increases in muscle glycogen stores, reduced plasma glycosylated hemoglobin levels and increases in fat free mass. This shows that resistance exercise is also a viable method for producing favorable changes in body composition but also improving the insulin signaling pathway, independent of increases in lean body mass. A subsequent study found that resistance training increases markers of IS and glycemic control that is independent of changes in muscle mass in T2D men and women (124). They found that resistance exercise of no more than 30 min in duration, three times per week increased GLUT4 protein content, insulin receptor content and glycogen synthase content (124). The combination of protein supplementation and resistance exercise also have a synergistic affect in diabetic populations. When a high protein diet and a resistance training program 3 times per week was combined in T2D men and women there was an approximate 2-fold reduction in insulin concentrations compared to control groups (70). However, the synergistic effects of a combined high protein and resistance training warrant future research in diabetic populations as it seems to be the most effective strategy to simultaneously increase lean body mass, decrease total body weight, and improve markers of glucose control independent of weight loss and thus may ultimately have the greatest effect in older adults at risk of developing or with PD/T2D.

## Conclusions

With age there is a loss of muscle mass and the development of IR, increasing the risk of sarcopenia and T2D. When these two conditions coincide, they can create a vicious cycle whereby IR induces greater muscle mass loss, leading to a further reduction in IS and vice versa. Protein has emerged as a potential strategy to combat the decline in muscle mass and IS that occur with increasing age, potentially preventing the development of T2D and sarcopenia. However, protein intake recommendations in older adults are currently insufficient at 0.8 g/kg/day, despite many groups advocating for increased requirements of 1.0–1.2 g/kg/day in older adults and 1.2–1.5 g/kg/day in those who are at risk of malnutrition. While trials in older, IR/PD/T2D populations are lacking, the evidence to date does support a role for higher protein intakes to attenuate declines in muscle mass and IS, particularly when combined with resistance exercise. Further work examining the effectiveness of higher protein intakes, with and without resistance training, in older adults with IR to prevent the development of sarcopenia and T2D are warranted.

## Author Contributions

MD and KB conceived, wrote, and edited the manuscript.

### Conflict of Interest Statement

The authors declare that the research was conducted in the absence of any commercial or financial relationships that could be construed as a potential conflict of interest.
